# Applying Existing Particle Paradigms to Inhaled Microplastic Particles

**DOI:** 10.3389/fpubh.2022.868822

**Published:** 2022-05-30

**Authors:** Stephanie Wright, Paul J. A. Borm

**Affiliations:** ^1^Environmental Research Group, Medical Research Council Centre for Environment and Health, School of Public Health, Imperial College London, London, United Kingdom; ^2^Nanoconsult, Meerssen, Netherlands

**Keywords:** microplastic, particle toxicology, inhalation [MeSH], exposure, physicochemical properties, particulate matter

## Abstract

Ambient particulate pollution originating from plastic contaminates air, including indoor and urban environments. The recent discovery of ambient microplastic (MP) particles of a size capable of depositing in the thoracic region of the airway, if inhaled, has raised concern for public exposure and health impacts following lessons learned from other particle domains. Current microplastic exposure estimates are relatively low compared to total ambient particulate matter, but optimal analytical techniques and therefore data for risk and health impact assessments are lacking. In the absence of such an evidence base, this paper explores paradigms, metrics and dose-response curves developed in other particle domains as a starting point for predicting whether microplastic are of concern. Bio-persistence, presence of reactive sites and soluble toxicants are likely key properties in microplastic toxicity, but these are not measured in environmental studies and hence are challenging to interpret in exposure. Data from a MP inhalation study in rats is available but the study was conducted using conditions that do not replicate the known human health effects of PM_2.5_ or surrogate exposures: compromised, aged animal models are recommended to investigate potential parallels between MPs and PM_2.5_. One of these parallels is provided by tire wear particles (TWP), which form part of current ambient PM and are sometimes regarded as microplastic. A connection to epidemiological studies where PM filters are still available is recommended and consequently analytical advances are required. In summary, established particle domains and existing paradigms provide valuable insight and data that can be used to predict MP toxicity, and direct study design and key properties to consider in this emerging field.

## Introduction

Microplastic particles (MPs) are comprised of diverse synthetic organic polymeric materials, varying by shape, size, density, weathering state, and chemical and/or microbial load. They originate from a variety of sources (plastic items and synthetic textiles) and activities and contaminate all of Earth's spheres. In air, their concentrations can range from <1 to 1,000 s per cubic meter (m^3^). The most frequently observed polymers in air are polyethylene and polyethylene terephthalate, with polypropylene, polystyrene, polyamide, and epoxy resin particles also commonly observed. Most studies find that fragments are the predominant shape, followed by fibers, however, all these variables depend on the sample type, environment, geographical location, and analytical methodology employed (1–4).

Another source of synthetic polymeric aerosols is non-exhaust vehicle emissions. Non-metallic and semi-metallic brake pads may include synthetic polymers in the composite material, and the majority of a (synthetic) tire is comprised of synthetic polymer (e.g., styrene butadiene styrene). Several studies estimate that 3–7% of the particulate air pollution fraction <2.5 μm aerodynamic diameter (PM_2.5_) consists of tire and brake wear [review: (5)] but there is considerable debate over whether these particles should be considered MPs (6). Many authors refer to tire wear particles (TWP) as MP due to its (semi-) synthetic polymer structure, solid state, insolubility, and particle size range (i.e., <1,000 μm). In the environment, however, pure TWP are rarely found. Instead, hybrid particles consisting of tire and road wear particles are present (7).

Irrespectively, MPs form part of the complex and dynamic particulate matter (PM) exposure profile. Acute and long-term exposure to the PM_2.5_ fraction is associated with increased mortality in cardiovascular and respiratory diseases (8, 9). Both PM_2.5_ and PM_10_ are associated with increased mortality from all causes, cardiovascular disease, respiratory disease, and lung cancer. Associations remain below the current WHO guideline exposure level of 10 μg/m^3^ for PM_2.5_ (8). Since the first study on the link between acute mortality and PM_2.5_ (10), several hundred studies have been published which have led to the estimate that an increase of 10 μg/m^3^ PM_2.5_ increases the risk of (premature) mortality by 1% (9).

Although MPs are claimed to be abundant in the environment, few studies are available that allow for an estimation of their relative contribution to PM_2.5_ or PM_10_ exposure, hence the potential role of MP in premature mortality and non-communicable disease is poorly understood. Irrespective of this, within environmental and occupational particle exposures, there are sub-fractions that are likely to be more harmful than others, such as transition metals in PM_10_ (11–13), which contributes very little to mass. Here, the concentration of transition metals can be seen as the biologically effective dose (BED) [review: (14, 15)]. It is therefore important to determine whether inhaled MPs share a common Mode of Action (MoA) with other inhaled particles or are of disproportionate concern and why. This paper explores the application of existing particle paradigms to MP in the lung environment and, more specifically, investigates the hypothesis that its action may be already part of the ambient particulate air pollution.

## Principles in Particle Toxicology

Over the past 50 years, scientific principles of particle toxicology have been developed in important particle domains reflecting occupational and, later, environmental exposures. Research in coal mine dust (16, 17), respirable crystalline silica (18), asbestos and man-made mineral fibers (19, 20), ambient PM, poorly soluble low toxicity particles such as TiO_2_ (21, 22), and engineered nanomaterials (23, 24) has helped define the physicochemical properties and dosimetric factors that influence the pathways leading to particle-triggered adverse outcomes (summarized in Table 1). The most important properties and factors are also interconnected. For example, the crucial “retained dose” in the lung following inhalation is equivalent to deposition (dependent on dimension and shape) minus what has been eliminated by defense (macrophage clearance) and/or dissolution (durability). Whilst all properties are important, some are more important in specific particle domains, such as shape/dimension and bio durability in the fiber domain and surface area in the engineered nanomaterials domain.

**Table 1:** Parameters and factors that play a major role in the biological response upon particle inhalation.

**Parameter**	**Description**	**Relevance for**
Durability	Biopersistence, dependent on defense as well as on particle properties (dissolution).	Fibers, PSLT
Density	The density of material, along with size and shape, determines aerodynamic behavior and deposition in the airway.	All
Size/Shape	Size distribution (diameter, length) and shape influence aerodynamic diameter along with density, and macrophage clearance.	AlL, but fibers specifically
Surface area	The quantitative surface available for interaction with the environment.	PSLT, nanomaterials
Chemical composition	Bulk composition is not equal to surface chemistry. In addition, toxic components may be released upon dissolution.	
Surface charge	The presence and composition of functional groups.	Positively charged particles (nylon flock, TWP)
Surface reactivity	Reactive groups and radicals on the surface	Crystalline silica
Dose	Cumulative dose for chronic effects; can be based on particle or fiber mass, number, or surface area. Bulk composition is not equal to surface chemistry.	All
Deposition	Dependent on dimension/shape and density, but also on airway morphology (hot spots).	All
Defense	Mucociliary clearance, macrophage clearance, inflammatory cells. If macrophage clearance is saturated, overload occurs; dose increases exponentially with time.	All
Retained dose	Dose retained in the lung after clearance and dissolution (determined by dose, defense and durability).	All

*PSLT, poorly soluble low toxicity particles; TWP, tire wear particles*.

In the concept of BED, surface, composition, and size/dimension are integrated, where intrinsic reactivity is a multiplier of surface area, depending on the material the particle is made of. In addition, lessons from asbestos and manmade vitreous fibers (MMVF) inform us that, for fibers, length is important and so “shape” needs to be included as a factor. Furthermore, studies on PM_10_ welding fumes, etc., have shown that many particles are complex, containing soluble components that can have considerable toxic potential. Finally, the duration the particle is likely to persist and avoid clearance determines the length of time that the BED is applied to the biological system (see “Durability,” Table 1); thus, a bio persistence factor is required (14). Taking these factors together, the paradigm that could predict the toxicity of an unknown particle consists of three main attributes for the biologically effective dose:

1. Surface attribute = surface area (SA) × specific surface reactivity (i.e., reactivity per unit SA) × surface availability (not all surface area is available for biological interaction)
2. Dimension attribute = length – diameter (mainly length if greater than a critical length), and when combined with density, influences aerodynamic behavior, deposition and thus exposure
3. Composition attribute = volume × specific volumetric reactivity (i.e., the toxic material per unit volume) × availability (= release rate i.e., amount per unit time)

It is logical to assume that the effects of inhaled MPs in the airway are driven by the same set of particle properties. The challenge is to find those properties that are crucial to describe and regulate its effects along the concept of BED.

## Microplastic Inhalation Exposure

Of the published studies on airborne MP which employed an analytical technique capable of distinguishing between plastics to calculate concentrations, just 10 were found for urban and indoor environments. These environments are considered most relevant to population exposure due to population density and the proportion of time spent indoors, and hence were used. The key details of these studies and characteristics of observed MP are summarized in Supplementary Table 1. The study by Soltani et al. (25), whilst on indoor deposition rather than air, calculates an inhalation exposure concentration estimate and is thus included. Most studies have focused on outdoor air, with 30% analyzing indoor air and 20% analyzing both outdoor and indoor samples.

Polyethylene and polyester/polyethylene terephthalate were equally found to be the most common polymers in studies. One of these studies only targets polycarbonate and polyethylene terephthalate (26) and therefore may not be representative of all polymers present. The study which focused on an emission source (synthetic nails/nail salon) found the acrylic nail material to be most common in both indoor and outdoor air (27). Eighty per cent of the studies found fragments and irregular shapes to be most common, not fibers as are often predicted.

Observed concentrations generally increased with increased instrument limits of detection, as expected. Higher concentrations were on the order of 100 s (2–4) to 1,000 s (1, 2) of MPs per cubic meter of air and most studies found the majority of particles to be distributed in the smaller size classes. Few studies explicitly estimate exposure. Chen et al. (28) estimate nail salon employees to be exposed to 260 MPs per day. However, if inhaled, these particles are likely to deposit in the upper airways and be swallowed due to the particle sizes being >25 μm. Just two studies used a method with a limit of detection below 10 μm (1, 4), which is relevant to human exposure in the central and lower airways and subsequent adverse health outcomes. In impactor samples (>2.5 μm and 2.5–1.0 μm aerodynamic diameter) collected near Bremen City, Germany, Kernchen et al. (4) found 67% of observed MP were below 10 μm in size, giving an exposure concentration of 60/m^3^. If normal adult minute ventilation is between 5 and 8 liters per minute, this results in an inhalation exposure estimate of 432–691 MPs per day. In a PM_10_ sample collected from an urban roadside site in London, UK, Levermore et al. (1) found 52% of observed MP were between 5 and 10 μm in size, resulting in an inhalation exposure estimate of 9,367–14,988 MPs per day. Whilst both studies adopt similar analytical methods and observe comparative proportions of <10 μm size classes and polyethylene, they are collected in different environments, which could explain the order of magnitude difference in concentration. Using high-density polyethylene (0.97 g/cm^3^) as a worst-case representative polymer from these studies, the volume of a 10 μm sphere, and the highest particle number exposure estimates for each study, worst-case mass concentration inhalation exposure estimates are 0.67–14.54 μg/day. It is important to highlight that those studies which do not analyze an aerodynamic fraction, collected by an aerodynamic size selective sampler, observe a very broad and coarse size distribution. Those studies which analyze a sample collected with an aerodynamic size selective sampler (1, 4) observe a narrower size distribution, with a modal size around 10 μm (1).

Some data is emerging to suggest MP is inhaled into the lung, through the analysis of lung tissue digestates (27, 29). Most of the MP observed in these studies are relatively large compared to what is expected (i.e., a median size of 10 μm or below). Whilst there is a small chance that these particles were inhaled from the environment, one would expect to see a greater abundance of smaller MP for the anatomical regions studied and hence more data is needed to draw conclusions from these observations. Additionally, *in situ* analyses, such as via spectral imaging, will strengthen this, such as the method applied by Chen et al. (28) which detected a cellulose fiber in a lung tumor tissue section.

In general, exposure to ambient MPs is largely unknown and has possibly been either underestimated or overestimated due to analytical challenges and sampling methodologies that are often incompatible with routine ambient PM sampling. The assessment of MPs in PM filters is a first step toward assessment of the respirable fraction in ambient air. There is still a great body of work to do to understand the size distribution of airborne MP, which requires physicists, chemists, engineers, and environmental scientists. Furthermore, whilst properties such as size, shape/dimension and bulk composition are often measured and reported, other metrics intrinsic to a structure-activity paradigm, such as the concentration of soluble impurities, presence of reactive sites or surface area are not. Laboratory-based experiments can begin to rank the toxicological importance of the various properties of MP, however, without knowledge on how these relate to environmental exposures, one cannot conclude the level of risk.

## Particle Bio-Persistence and Adverse Outcomes

An array of lung cells generate inflammatory mediators and intracellular ROS upon exposure to diesel exhaust particles and PM (30) and also to MP model particles (31, 32). *In vivo*, acute inflammation is immediately followed by resolution and tissue repair, mediated through specialized pro-resolving mediators (SPMs) and type 2 cytokines and cells, including M2 macrophages and Th2 lymphocytes. For biopersistent particles and fibers in the lung, dose-dependent inflammation can progress to a type 2 inflammation, which eventually promotes interstitial fibrosis, granuloma formation, and tumorigenesis (reviewed in: 24). Recent studies also reveal the involvement of regulatory T-cells in the subsequent pathogenesis caused by inhaled particulates and therefore a long-term immune response is considered to be part of the long-term outcome (33). The persistence of MPs is thus considered a highly relevant property to consider in MP toxicology. Synthetic fibers (polypropylene, polyethylene, and polycarbonate) showed no dissolution, no significant changes to surface area and very slight weight gain following a 180 day *in vitro* leaching test in physiological fluid (Gamble's Solution), suggesting they may persist *in vivo* (34). However, respirable para-aramid fibers are considered a low risk since they have been observed to shorten in the lung and undergo rapid clearance in *in vivo* experimental studies. It has been hypothesized that lung fluid coats the fibers, catalyzing enzymatic attack and enabling biodegradation of inhaled p-aramid fibers in the lungs (35). Degradation has also been observed for inhaled polypropylene fibers in rats, which increased with exposure concentration and time (36). Whether this is apparent for other synthetic fibers is largely unknown and experiments are needed to assess their solubility, considering pH and enzymes. What is noteworthy, is that cellulose fibers were found to be more persistent than p-aramid fibers *in vivo* (37), and thus manmade cellulose fibers should be included in synthetic fiber hazard assessments. The inhalation of elevated levels of respirable plastic dust in occupational settings has been linked to interstitial lung diseases (38). Whether this pathogenicity is due to biopersistence and particle overload or the positive charges on the nitrogen atoms of nylon flock (39) remains untested.

Given the substantial variation within and between particle domains and MPs in their size, shape, aspect ratio, rigidity, and other physicochemical properties, it is rational to assume that their interaction with the immune system in the lung will differ within and from one another. The physicochemical properties of a particle govern the composition of the protein corona it acquires in biological fluids, which in turn affects bioavailability and fate *in vivo*. Since these properties vary by polymer, it is difficult to predict the behavior of a material *in vivo* (40). However, there are common disease phenotypes among different particles, fibers, and nanomaterials (Table 2). Therefore, whilst the molecular initiating events may differ, it is likely that some particulates share a similar adverse outcome pathway, such as inflammation > chronic inflammation and aberrant repair > fibrosis (33).

**Table 2:** Typical exposures, health outcomes, and current exposure standards for different particle domains.

**Particle group**	**Main health effects**	**Exposure characteristics**	**Material characteristics**
Coal mine dust	Lung function decrease, bronchitis, CWP, PMF, emphysema	Occupational, underground and surface (coal) mining 2–40 mg/m^3^ for up to 20 years	Mixtures of minerals (crystalline silica) and organic components. Current OEL: 4 mg/m^3^
Asbestos mineral fibers	Lung function decrease, lung cancer, mesothelioma	Occupational and consumers, insulation and production 1–1,000 fibers/cc	Fiber shaped minerals. Standards around 1 fiber /cc in most countries, long (>15 μm) and thin (<3 μm) of greatest concern.
Poorly soluble low toxicity particles (PSLT)	Lung function decrease, fibrosis, cancer	Occupational exposure (nuisance dusts). OEL values between 4 and 10 mg/m^3^	Diverse group of insoluble materials including polymers, CB, TiO_2_, talc, toner pigments.
PM_2.5_/PM_10_	Increased acute mortality and morbidity in patients with COPD or cardiovascular problems, long term cause of diabetes/lung cancer	Environmental exposure. WHO exposure standard: up to 100 μg/m^3^ (24 h)	Complex mix of many components and adsorbed compounds varying per time and space. Includes ultrafine particles. Current standard: 20 μg/m^3^ (24 h). No standard for UF particles.
Nanomaterials	No general health effects indicated	Occupational and consumer exposure (particle numbers and surface area instead of mass)	Endless variability in size, surface chemistry, and sub-molecular properties, with at least one dimension measuring <100 nm. Standards available for subtypes (TiO_2_, CNT)
Microplastic particles	No general hazard identified	Omnipresent at low levels of exposure. Mainly non-respirable (>50 μm) due to analytical limitations	Synthetic and semi-synthetic materials, usually fibrous and fragments. No standard available

*CWP, coal worker's pneumoconiosis; PMF, Progressive massive fibrosis; COPD, chronic obstructive pulmonary disease; CNT, carbon nanotubes; OEL, occupational exposure limit; MAK, German commission for occupational exposure limits; UF, ultrafine*.

## Inhalation Studies With Microplastic Particles or Surrogates

Despite being a recognized component of PM for longer, whether ambient tire wear proportionally contributes to the effects of PM_2.5_ is still being determined. Available *in vivo* toxicity data, obtained using TWP collected at a road simulator laboratory, suggest that inhaled TWP exert only mild respiratory toxicity (41). Female Sprague Dawley (SD) rats (*n* = 10/treatment group) were exposed to TWP at 10, 40, or 100 μg/m^3^ via nose-only inhalation for 6 h/day for 28 days and toxicity was assessed following OECD guidelines (TG 412). No TWP-related effects were observed on survival, clinical observations, body or organ weights, gross pathology, food consumption, immune system endpoints, serum chemistry, or biochemical markers of inflammation or cytotoxicity (41). Unfortunately, PM_2.5_ was not used as a reference, although this was part of the study hypothesis, and only diesel exhaust particles and mineral particles such as TiO_2_ and SiO_2_ were used in a separate study-arm investigating bronchoalveolar lavage (BAL) after intratracheal instillation (41). Based on the NOAEL level from the TWP study (55 μg/m^3^), and exposure estimates for TWP, this group of investigators propose that a margin of exposure of 400–700 is present between the current PM_2.5_ standard and TWP exposure (42, 43).

Important to note is that health effects due to ambient PM exposure are observed mainly in sensitive subgroups within human populations and in animal models mimicking such populations (44). Nearly all reported *in vivo* studies on toxicological responses to ambient PM and/or standards produced no or low responses in normal laboratory animals compared to effects seen in humans. Therefore, spontaneously hypertensive (SH) rats and APO E^−/−^ deficient mice have been used to model acute respiratory and cardiovascular responses to ambient PM (see Supplementary Table 2), whereas OECD protocols prescribe the use of normal rats or mice.

A selection of animal inhalation studies with ambient PM or surrogates have been included in Supplementary Table 2 to illustrate that responses in compromised animals mimic cardiovascular effects such as blood pressure, heart rate variability and systemic inflammation observed in humans in epidemiological studies. However, the concentrations needed to induce systemic effects with ambient air particles are usually higher in (compromised) rats than in man (Supplementary Table 2). As a benchmark, intratracheal instillation of 1,000 μg, but not 200 and 500 μg, PM_2.5_ suspension in aged SHR rats led to pulmonary and systemic events (45), whereas instillation of 100 μg of PM_2.5_ suspension in humans led to significant cellular responses in the BAL fluid (11). This underscores the fact that no current animal models reflect human sensitivity to inhaled fine particles.

Therefore, it is no surprise that studies with normal animals, according to OECD protocols, have shown little effects for higher concentrations of polymer, tire and road wear particles (see Supplementary Table 2) and pharmaceutical acrylic ester polymers (data not shown). However, rats exposed to polystyrene nanoparticle mass concentrations similar to those of TWP (20, 50 and 100 μg/m^3^) for 6 h per day, 5 days/week for 2 weeks showed a statistically significant decrease in hematological parameters, including white blood cell and lymphocyte counts, and a statistically significant increase in percent and number of eosinophils, but only in female rats (32). Effects on lung lavage were less pronounced, due to high variation, but a potential inflammatory and fibrotic response was observed from the lowest concentration of PS nanoparticles at 22 μg/m^3^ (32). The retained dose per rat (5–34 μg/day) (32) is similar to the alveolar burden in ultra-fine (50 nm) carbon particle studies (45, 46), estimated at 10.6 μg or 5.5 × 10^11^ ultra-fine carbon particles. It is evident that MP inhalation studies (Supplementary Table 2) used longer exposure times than used for PM_2.5_ and focused on sub-chronic effects in the lung; however, the exposure concentrations used are not dissimilar from the PM studies in compromised animals. Thus, a retained dose-based read across is plausible for the inflammatory outcomes in the lung. Effects in compromised animals remains to be studied.

## Exposure to Particle-Chemical Mixtures

In both PM_2.5_ and PM_10_ epidemiological studies, black carbon has been identified as a separate descriptor of health effects (47, 48) and reduced exposure is recommended. Whilst not directly toxic, toxicological studies suggest that black carbon may operate as a universal carrier of toxic species, providing a mechanism of entry to the human body and transport to target tissues. Low concentrations of components absorbed on the particle surface may actually be the driver of the adverse response. In this context it has been shown that metals and quinones on the particles surface are crucial for initiating an inflammatory response (11) in humans and DNA damage (49) in rats exposed to PM. In addition, bacterial endotoxin (LPS), which is a well-known initiator of inflammatory cascade, is commonly adsorbed on PM, although most prominent in PM_10_ and less in PM_2.5_ (50).

Such a carrier mechanism can be easily assumed for substances absorbed on or contained in MPs, following inhalation. Several different synthetic polymers have been shown to carry different toxic metals (51, 52), including redox-active Cu-ions. The absorption of metals on MPs has also been shown to affect the kinetics and toxicity of both metals and particles in *Daphna magnia* (53) and Zebrafish embryos (54), but little data using human target cells are available. Bradney et al. (55) discussed the potential mechanisms and evidence by which trace elements (including metals) can achieve a different ADME when carried by MP but had no specific outcome for inhaled MPs. A recent review by Hahladakis et al. (56) provides an overview of all potential chemical additives present in plastics. While this overview focused on migration, release and fate of additives, no attention is given as to the additive effect of such components to particle toxicity. From other particle domains, including PM, we know such can be a very important trigger of the inflammatory response such as through redox mechanisms or surface reactivity (49). It also needs to be recognized that in some cases the role of contaminants has been overestimated. Well-designed inhalation studies showed that the role of polycyclic aromatic hydrocarbons carried by diesel exhaust particles was much less important for toxicity than initially assumed (57, 58).

The absorption of external contaminants on airborne MPs is an area which requires research and modeling. Thermodynamic modeling has shown that at least in the aquatic environment, MP contributes a negligible amount of chemicals to biota, relative to the environment and uptake via natural pray, mostly due to their low concentrations and therefore low likelihood of an organism encountering one relative to pray (59, 60). Studies in ambient PM have shown that particles aggregate and agglomerate during formation and transport and particle composition is determined by condensation and colliding and merging of primary and secondary particles even during the day (61). Based on these observations and the occurrence of many particle types that can be considered as MPs (e.g., tire wear, fibrous fragments) studies are needed to evaluate whether ambient MPs may act as a vector or form particle complexes, which increase exposure and may enhance particle activity, such as for ambient PM. This should be considered for total suspended MP, since inhaled MP > 10 μm in aerodynamic diameter may still deposit in the upper airways.

## Considering Microplastic As Part Of PM_2.5_

Another approach to explore potential MP-driven health effects is to study the MP content in PM_2.5_ or PM_10_ filters of exposure studies that have been used in epidemiological studies. Levermore et al. (1) showed that both virgin and environmental MPs (>2 μm) can be detected via Raman spectral images. Application of this technique to available filter samples could give an estimate on the percentage of MP in those samples for comparison with available estimates (42, 43). The health impact of that presence could be calculated via the proportion of PM_10_ or PM_2.5_ and associated health risks (top-down approach) or via the rat toxicity data of TWP (42) or model polymers [e.g., (32)], which can be considered as bottom-up. The top-down approach would be based on many samples from all over the globe that are linked to both acute and chronic mortality due to PM. The assumption that the effect is based on the mass or number proportion is simply based on assuming that the best metric of health effects of PM is mass/m^3^.

As an example, if 0.1% of the mass on PM filters is microplastic, the proportionate mortality rate at 10 μg/m^3^ increase would be 1%/1,000 = 0.001%, instead of 1% as observed for PM_2.5_ (9). Even though this number is small, the recent analysis by Schwartz et al. (62) on individuals continually exposed to low PM levels found this led to 14,000 premature deaths per year per 1 μg/m^3^ in the USA. Extrapolating this to MP (and 0.1 % of mass) would still lead to significant numbers at the current average PM exposure. Of course, this is with limitations, since it assumes proportional linearity in the toxicity of different PM sources, but it provides a start.

In summary, MPs likely have similar hazards to other particle domains, such as ambient PM, PSLTs and engineered nanomaterials. However, in the absence of inhalation exposure assessments, the level of risk is uncertain. Either way, plastic is a source of PM_10_ and PM_2.5_, which are regulated on a (total) mass basis only. Thus, a better understanding of MP sources and quantification of emissions will determine whether and where interventions are needed to ultimately reduce PM_10/2.5_ exposure and future disease burdens. The concept of BED provides a conceptual basis for read-across. The concept may provide a framework for filling in knowledge gaps for MP such as (gravimetric) exposure, particle dimensions and surface activity in lung response.

## Author Contributions

SW and PB contributed equally to the conception and design of the article, the acquisition, evaluation and interpretation of the data and the drafting and editing of the manuscript. Both authors contributed to the article and approved the submitted version.

## Funding

SW was supported by the Medical Research Council (MRC) Centre for Environment and Health and is a member of the Health Protection Research Unit in Environmental Exposures and Hazards, a partnership between UK Health Security Agency (UKHSA) and Imperial College London which was funded by the National Institute for Health Research (NIHR). The views expressed are those of the author and not necessarily those of the MRC, NIHR, and UKHSA. PB is consultant toxicologist at Nanoconsult and professor in Particle Toxicology at University of Dusseldorf, and the work that led to this paper was funded by the International Council of Chemical Associations (ICCA). Nanoconsult is also a partner of the Dutch Zon-MW program MOMENTUM.

## Conflict of Interest

PB is managing director of Nanoconsult and, in this function, has received funding from ICCA to conduct a microplastic read-across analysis. However, the outcomes of this analysis are not congruent to the vision of ICCA. The remaining author declares that the research was conducted in the absence of any commercial or financial relationships that could be construed as a potential conflict of interest.

## Publisher's Note

All claims expressed in this article are solely those of the authors and do not necessarily represent those of their affiliated organizations, or those of the publisher, the editors and the reviewers. Any product that may be evaluated in this article, or claim that may be made by its manufacturer, is not guaranteed or endorsed by the publisher.

## References

[1] Levermore JM Smith TEL Kelly FJ Wright SL. Detection of microplastics in ambient particulate matter using raman spectral imaging and chemometric analysis. Anal Chem. (2020) 92:8732–40. 10.1021/acs.analchem.9b05445 32568507

[2] Liao Z Ji X Ma Y Lv B Huang W Zhu X. Airborne microplastics in indoor and outdoor environments of a coastal city in Eastern China. J Hazard Mater. (2021) 417:126007. 10.1016/j.jhazmat.2021.126007 33992007

[3] Zhu X Huang W Fang M Liao Z Wang Y Xu L. Airborne microplastic concentrations in five megacities of Northern and Southeast China. Environ Sci Technol. (2021) 55:12871–81. 10.1021/acs.est.1c03618 34559513

[4] Kernchen S Löder MGJ Fischer F Fischer D Moses SR Georgi C. Airborne microplastic concentrations and deposition across the Weser River catchment. Sci Total Environ. (2021) 818:151812. 10.1016/j.scitotenv.2021.151812 34808158

[5] Baensch-Baltruschat B Kocher B Stock F Reifferscheid G. Tyre and road wear particles (TRWP) - A review of generation, properties, emissions, human health risk, ecotoxicity, and fate in the environment. Sci Total Environ. (2020) 733:137823. 10.1016/j.scitotenv.2020.137823 32422457

[6] Ecetoc. The ECETOC Conceptual Framework for Polymer Risk Assessment (CF4Polymers) Technical Report No. 133-1, Brussels (2019).

[7] Sommer F Dietze V Baum A Sauer J Gilge S Maschowski C. Tire abrasion as a major source of microplastics in the environment. Aerosol Air Qual. Res. (2018) 18:2014–28. 10.4209/aaqr.2018.03.0099

[8] Chen J Hoek G. Long-term exposure to PM and all-cause and cause-specific mortality: a systematic review and meta-analysis. Environ Int. (2020) 143:105974. 10.1016/j.envint.2020.105974 32703584

[9] WHO. Ambient (Outdoor) Air Pollution. (2021). Available online at: https://www.who.int/news-room/fact-sheets/detail/ambient-(outdoor)-air-quality-and-health

[10] Dockery DW Pope CA III Xu X Spengler JD Ware JH Fay ME. An association between air pollution and mortality in six U.S. cities. N Engl J Med. (1993) 329:1753–9. 10.1056/NEJM199312093292401 8179653

[11] Schaumann F Borm PJ Herbrich A Knoch J Pitz M Schins RP. Metal-rich ambient particles (particulate matter2. 5) cause airway inflammation in healthy subjects. Am J Respir Crit Care Med. (2004) 170:898–903. 10.1164/rccm.200403-423OC 15229099

[12] Molinelli AR Madden MC McGee JK Stonehuerner JG Ghio AJ. Effect of metal removal on the toxicity of airborne particulate matter from the Utah Valley. Inhal Toxicol. (2002) 14:1069–86. 10.1080/08958370290084737 12396411

[13] Jiménez LA Thompson J Brown DA Rahman I Antonicelli F Duffin R. Activation of NF-kappaB by PM(10) occurs via an iron-mediated mechanism in the absence of IkappaB degradation. Toxicol Appl Pharmacol. (2000) 66:101–10. 10.1006/taap.2000.8957 10896851

[14] Donaldson K Tran L. Borm PJA. The toxicology of inhaled particles: summing up and emerging conceptual framework. In: Donaldson and Borm, editors. Particle Toxicology. Boca Raton, FL: Francis (2007) p. 413–24. 10.1201/9781420003147.ch22

[15] Schulte PA Rothman N Hainaut P Smith MT Boffetta P Perera FP. Molecular epidemiology: linking molecular scale insights to population impacts. IARC Sci Publ. (2011) 1–7. 22997853

[16] Hurley JF Alexander WP Hazledine DJ Jacobsen M Maclaren WM. Exposure to respirable coalmine dust and incidence of progressive massive fibrosis. Br J Ind Med. (1987) 44:661–72. 10.1136/oem.44.10.661 3676119 PMC1007898

[17] Seaton A. Farewell, King Coal: From Industrial Triumph to Climatic Disaster. Dunedin Academic Press Ltd., Edinburgh-London (2018).

[18] Pavan C Fubini B. Unveiling the variability of “quartz hazard” in light of recent toxicological findings. Chem Res Toxicol. (2017) 30:469–85. 10.1021/acs.chemrestox.6b00409 27992190

[19] Mossman BT Gee JB. Asbestos-related diseases. N Engl J Med. (1989) 320:1721–30. 10.1056/NEJM198906293202604 2659987

[20] Oberdörster G. Determinants of the pathogenicity of man-made vitreous fibres (MMVF). Int Arch Occ Environ Health. (2000) 73:S60–8. 10.1007/PL00014628 10968563

[21] Driscoll KE Borm PJA. Expert workshop on the hazards and risks of poorly soluble low toxicity particles. Inhal Toxicol. (2020) 32:53–62. 10.1080/08958378.2020.1735581 32149535

[22] Morrow PE. Possible mechanisms to explain dust overloading of the lungs. Fundam Appl Toxicol. (1988) 10:369–84. 10.1093/toxsci/10.3.369 3286345

[23] Borm PJ Robbins D Haubold S Kuhlbusch T Fissan H Donaldson K. The potential risks of nanomaterials: a review carried out for ECETOC. Part Fibre Toxicol. (2006) 3:11. 10.1186/1743-8977-3-11 16907977 PMC1584248

[24] Riediker M Zink D Kreyling W Oberdörster G Elder A Graham U. Particle toxicology and health - where are we? Part Fibre Toxicol. (2019) 16:26. 10.1186/s12989-019-0308-2 31248442 PMC6595670

[25] Soltani NS Taylor MP Wilson SP. Quantification and exposure assessment of microplastics in Australian indoor house dust. Environ Poll. (2021) 283:117064. 10.1016/j.envpol.2021.117064 33862344

[26] Zhang J Wang L Kannan K. Microplastics in house dust from 12 countries and associated human exposure. Environ Int. (2020) 134:105314. 10.1016/j.envint.2019.105314 31756678

[27] Chen E-Y Lin K-T Jung C-C Chang C-L Chen C-Y. Characteristics and influencing factors of airborne microplastics in nail salons. Sci Total Environ. (2022) 806:151472. 10.1016/j.scitotenv.2021.151472 34742808

[28] Chen Q Gao J Yu H Yang Y Cao Y Zhang Q. An emerging role of microplastics in the etiology of lung ground glass nodules. Envrion Sci Eur. (2022) 34:25. 10.1186/s12302-022-00605-3

[29] Jenner LC Rotchell JM Bennett RT Cowen M Tentzeris V Sadofsky LR. Detection of microplastics in human lung tissue using μFTIR spectroscopy. Sci Total Environ. (2022) 831:154907. 10.1016/j.scitotenv.2022.154907 35364151

[30] Valavanidis A Vlachogianni T Fiotakis K. Loridas S. Pulmonary oxidative stress, inflammation and cancer: respirable particulate matter, fibrous dusts and ozone as major causes of lung carcinogenesis through reactive oxygen species mechanisms. Int J Environ Res Public Health. (2013) 10:3886–907. 10.3390/ijerph10093886 23985773 PMC3799517

[31] Hwang J Choi D Han S Choi J Hong J. An assessment of the toxicity of polypropylene microplastics in human derived cells. Sci Total Environ. (2019) 684:657–69. 10.1016/j.scitotenv.2019.05.071 31158627

[32] Lim D Jeong J Song KS Sung JH Oh SM Choi J. Inhalation toxicity of polystyrene micro(nano)-plastics using modified OECD TG 412. Chemosphere. (2021) 262:128330. 10.1016/j.chemosphere.2020.128330 33182093

[33] Ma Q. Polarization of immune cells in the pathologic response to inhaled particulates. Front Immunol. (2020) 11:1060. 10.3389/fimmu.2020.01060 32625201 PMC7311785

[34] Law BD Bunn WB Hesterberg TW. Solubility of polymeric organic fibers and manmade vitreous fibers in gambles solution. Inhal Toxicol. (1990) 2:321–39. 10.3109/08958379009145261

[35] Warheit DB Reed KL Pinkerton KE Webb TR. Biodegradability of inhaled p-aramid respirable fiber-shaped particulates (RFP): mechanisms of RFP shortening and evidence of reversibility of pulmonary lesions. Toxicol Lett. (2002) 127:259–67. 10.1016/S0378-4274(01)00508-2 12052666

[36] Hesterberg TW McConnell EE Miller WC Hamilton R Bunn WB. Pulmonary toxicity of inhaled polypropylene fibers in rats. Fundam Appl Toxicol. (1992) 19:358–66. 10.1093/toxsci/19.3.358 1459367

[37] Warheit DB Hartsky MA Reed KL Webb TR. Biodegradability of inhaled para-aramid respirable-sized fiber-shaped particulates: mechanistic *in vivo* and *in vitro* studies. Toxicol Appl Pharmacol. (2001) 174:78–88. 10.1006/taap.2001.9177 11437651

[38] Kern DG Kuhn C III Ely EW Pransky GS Mello CJ Fraire AE. Flock worker's lung: broadening the spectrum of clinicopathology, narrowing the spectrum of suspected etiologies. Chest. (2000) 117:251–9. 10.1378/chest.117.1.251 10631226

[39] Nemery B Hoet PH. Humidifier disinfectant-associated interstitial lung disease and the Ardystil syndrome. Am J Respir Crit Care Med. (2015) 191:116–7. 10.1164/rccm.201409-1726LE 25551352

[40] Fuchs AV Gemmella AC Thurecht KJ. Utilising polymers to understand diseases: advanced molecular imaging agents. Polym Chem. (2015) 6:868–880. 10.1039/C4PY01311E

[41] Kreider ML Doyle-Eisele M Russell RG McDonald JD Panko JM. Evaluation of potential for toxicity from subacute inhalation of tire and road wear particles in rats. Inhal Toxicol. (2012) 24:907–17. 10.3109/08958378.2012.730071 23121300

[42] Kreider M Unice KM Panko JM. Human health risk assessment of tire and road wear particles (TWP) in air. Human Ecol Risk Ass. (2020) 26:1–9. 10.1080/10807039.2019.1674633

[43] Panko JM Hitchcock KM Fuller GW Green D. Evaluation of tire wear contribution to PM2.5 in urban environments. Atmosphere. (2019) 10:99. 10.3390/atmos1002009924941699

[44] Lippmann M. Toxicological and epidemiological studies of cardiovascular effects of ambient air fine particulate matter (PM2.5) and its chemical components: coherence and public health implications. Crit Rev Toxicol. (2014) 44:299–347. 10.3109/10408444.2013.861796 24494826

[45] Upadhyay S Ganguly K Stoeger T Semmler-Bhenke M Takenaka S Kreyling WG. Cardiovascular and inflammatory effects of intratracheally instilled ambient dust from Augsburg, Germany, in spontaneously hypertensive rats (SHRs). Part Fibre Toxicol. (2010) 7:27. 10.1186/1743-8977-7-27 20920269 PMC2956709

[46] Upadhyay S Stoeger T George L Schladweiler MC Kodavanti U Ganguly K. Ultrafine carbon particle mediated cardiovascular impairment of aged spontaneously hypertensive rats. Part Fibre Toxicol. (2014) 11:36. 10.1186/s12989-014-0036-6 25442699 PMC4410795

[47] Janssen NA Hoek G Simic-Lawson M Fischer P van Bree L ten Brink H. Black carbon as an additional indicator of the adverse health effects of airborne particles compared with PM10 and PM2.5. Environ Health Perspect. (2011) 119:1691–9. 10.1289/ehp.1003369 21810552 PMC3261976

[48] Janssen NAH Gerlofs-Nijland ME Lanki T Salonen RO Cassee F Hoek G. Health Effects of Black Carbon WHO Report (2012).

[49] Knaapen AM Güngör N Schins RP Borm PJ Van Schooten FJ. Neutrophils and respiratory tract DNA damage and mutagenesis: a review. Mutagenesis. (2006) 21:225–36. 10.1093/mutage/gel032 16870698

[50] Schins RP Lightbody JH Borm PJ Shi T Donaldson K Stone V. Inflammatory effects of coarse and fine particulate matter in relation to chemical and biological constituents. Toxicol Appl Pharmacol. (2004) 195:1–11. 10.1016/j.taap.2003.10.002 14962500

[51] Godoy V Blázquez G Calero M Quesada L Martín-Lara MA. The potential of microplastics as carriers of metals. Environ Pollut. 255:113363. 10.1016/j.envpol.2019.113363 31614247

[52] Zou J Liu X Zhang D Yuan X. Adsorption of three bivalent metals by four chemical distinct microplastics. Chemosphere. (2020) 248:126064. 10.1016/j.chemosphere.2020.126064 32041068

[53] Yuan W Zhou Y Chen Y Liu X Wang J. Toxicological effects of microplastics and heavy metals on the Daphnia magna. Sci Total Environ. (2020) 746:141254. 10.1016/j.scitotenv.2020.141254 32768788

[54] Zhang R Wang M Chen X Yang C Wu L. Combined toxicity of microplastics and cadmium on the zebrafish embryos (Danio rerio). Sci Total Environ. (2020) 743:140638. 10.1016/j.scitotenv.2020.140638 32679492

[55] Bradney L Wijesekara H Palansooriya KN Obadamudalige N Bolan NS Ok YS. Particulate plastics as a vector for toxic trace-element uptake by aquatic and terrestrial organisms and human health risk. Environ Int. (2019) 131:104937. 10.1016/j.envint.2019.104937 31284110

[56] Hahladakis JN Velis CA Weber R Iacovidou E Purnell P. An overview of chemical additives present in plastics: migration, release, fate and environmental impact during their use, disposal and recycling. J Hazard Mater. (2018) 344:179–99. 10.1016/j.jhazmat.2017.10.014 29035713

[57] Nikula KJ Snipes MB Barr EB Griffith WC Henderson RF Mauderly JL. Comparative pulmonary toxicities and carcinogenicities of chronically inhaled diesel exhaust and carbon black in F344 rats. Fundam Appl Toxicol. (1995) 25:80–94. 10.1093/toxsci/25.1.80 7541380 PMC7528960

[58] Dasenbrock C Peters L Creutzenberg O Heinrich U. The carcinogenic potency of carbon particles with and without PAH after repeated intratracheal administration in the rat. Toxicol Lett. (1996) 88:15–21. 10.1016/0378-4274(96)03712-5 8920711

[59] Gouin T Roche N Lohmann R Hodges G. A thermodynamic approach for assessing the environmental exposure of chemicals absorbed to microplastic. Environ Sci Technol. (2011) 45:1466–72. 10.1021/es1032025 21268630

[60] Koelmans AA Bakir A Burton GA Janssen CR. Microplastic as a vector for chemicals in the aquatic environment: critical review and model-supported reinterpretation of empirical studies. Environ Sci Technol. (2016) 50:3315–26. 10.1021/acs.est.5b06069 26946978 PMC6863595

[61] Demerjian KL Mohnen VA. Synopsis of the temporal variation of particulate matter composition and size. J Air Waste Manag Assoc. (2008) 58:216–33. 10.3155/1047-3289.58.2.216 18318338

[62] Schwartz J Wei Y Yitshak-Sade M Di Q Dominici F Zanobetti AA. and national difference in differences analysis of the effect of PM2.5 on annual death rates. Environ Res. (2021) 194:110649. 10.1016/j.envres.2020.110649 33385394 PMC11003463

