# Supplementary material for: Applying Existing Particle Paradigms to Inhaled Microplastic Particles
Source: Front Public Health. 2022 May 30;10:868822. doi: 10.3389/fpubh.2022.868822 (PMC9197419; doi:10.3389/fpubh.2022.868822)
Supplement: Supplementary file 1 [file Table_1.DOCX]

| **Reference** | **Country** | **Environment** | **Environment Type** | **Sample** | **LOD** | **Concentration** | **Size Range (um)** | **Average Size (um)** | **Main Polymer** | **Main Shape** | **Inhalation Exposure** |
| --- | --- | --- | --- | --- | --- | --- | --- | --- | --- | --- | --- |
| Chen et al., 2022 | Taiwan | Indoor | Urban (nail salon) | TSP (low vol.), ~10 h | 25 um | 46/m3 | 25.0 to >200.0 | 25.0-50.0 (55%) | AC (27%) | Fragments (99%) | 260 ± 315 MPs/day (TSP) |
|  |  | Outdoor |  |  |  | 28/m3 |  | 25.0-50.0 (79%) | AC (40%) | Fragments (99%) |  |
| Amato-Lourenco et al., 2022 | Brazil | Outdoor | Urban (Medical Centre) | TSP (low vol.), 24 h | 50 um | 7-24/m3 | 50.0 to 1579.4 (fibres) | 162.0 ±1.6 (fibres) | PES (84%) | Fibres (~98%) |  |
|  |  |  |  |  |  | 0-1/m3 | 50.1 to 877.1 (particles) | 110.2 ±4.2 (particles) |  |  |  |
| Kernchen et al., 2021 | Germany | Outdoor | Urban (Weser river catchment) | TSP (low vol.), 3 h | 4 um | 121/m3 or 108 ng/m3 | 4.4 to 29.0 | <10.0 (67%) | PE (78%) | Fragment (79%) | 60/m3 (<10 um, 3 h) |
| Zhu et al., 2021 | China | Outdoor | Urban (megacities) | TSP (mid vol.), 1 m3 | 10 um | 282/m3 | 5.9 to 1475.3 | <30.0 (62%) | PE (26.6%) | Fragments (88%) |  |
| Liao et al., 2021 | China | Outdoor | Urban (Wenzhou City) | TSP (mid vol.), 1 m3 | 10 um | 189/m3 | 5.0 to 5000.0 | 5.0–30.0 (65%) | PE (26.8%) | Fragments (94.2%) |  |
|  |  | Indoor |  |  |  | 1583/m3 | 5.0 to 5000.0 | 5.0–30.0 (60%) | PES (28.4%) | Fragments (89.6%) |  |
| Soltani et al., 2021 | Australia | Indoor | Urban (Sydney) | Deposition (4 weeks) | 50 um | 3095 fibres/m2/day | 5.0 to 5000.0 | 200.0 to 400.0 l x 18.0 d (fibres) | PE (25%) | Fibres (99%) | 12891±4472 fibres/year (TSP) |
|  |  |  |  |  |  |  |  | 686.0 (fragments) |  |  |  |
|  |  |  |  |  |  |  |  | 100.0 (films) |  |  |  |
| Levermore et al., 2020 | UK | Outdoor | Urban (roadside, London) | PM10, 24 h | 2 um | 2502/m3 | 4.7 to 40.9 | 5.0-10.0 (52%) | PE (>99%) | Fragments (100%) | 1301/m3 (PM10) |
| Zhang et al., 2020 | Global | Indoor | Urban (residential) | Floor dust - vacuum or sweeping | 5.3 (PET), 0.11 µg/g (PC) | 25,000 µg/g (PET - Japan) | - | - | PET (>99%) | - | 360–150,000 ng/kg-bw/day (PET) |
|  |  |  |  |  |  | 45 µg/g (PC - Saudi Arabia) | - | - |  | - | 0.88–270 ng/kg-bw/day (PC) |
| Liu et al., 2020 | China | Outdoor | Urban | TSP, 1 to 144 m3 | N.R. | 0.41/m3 | 12.4 to 2191.3 | 246.5 | PET (51%) | Fragments (48%) |  |
| Vianello et al., 2019 | Denmark | Indoor | Urban (residential) | Breathing Thermal Manikin, 24 h | 11 um | 9.3 ± 5.8/m3 | 11.0 x 5.5 | 36.0 x 21.0 | PES (81%) | Fragments (87%) | 9.3 ± 5.8 (TSP, 24 h) |

**Supplementary Table 1.** Summary of the characteristics of airborne microplastic observed in air in urban and/or indoor environments. Search term ‘microplastic AND air’. Inclusion criteria: primary research, quantifying microplastic in a volume of air, uses an analytical technique which can discriminate between plastic types and uses this data to estimate concentration.
