# Supplementary material for: Applying Existing Particle Paradigms to Inhaled Microplastic Particles
Source: Front Public Health. 2022 May 30;10:868822. doi: 10.3389/fpubh.2022.868822 (PMC9197419; doi:10.3389/fpubh.2022.868822)
Supplement: Supplementary file 2 [file Table_2.DOCX]

| Particle type | Concentrations/exposure | Outcome | Animal type | Study |
| --- | --- | --- | --- | --- |
| **Microplastic particles** | | | | |
| Polystyrene (100 nm) | 21, 43, and 100 μg/m³, 6 h/day, 5 d/week, 2 weeks | Decrease white blood cells, increase lung inflammation at all concentrations. | Female and male SD rats | Lim et al (2021) |
| Tyre and road wear particles (2.4 – 3.7 um) | 10, 40, and 100 μg/m³, 6 h/day, 28 days | Neutrophil increase at 10, but not at 40 and 100 μg/m^3^. Slight dose-dependent increase in total cells. | Female and male SD rats | Kreider et al (2012) |
| **Ambient PM and surrogates** | | | | |
| Ultrafine carbon particles (UfCP)  Ultrafine carbon particles (UfCP) | 24 h (~180 μg/m³)  24 h (~172 μg/m³) | Increased BP and HR. Increase in neutrophils and IL-6 in BAL. Cardiac BP and HR in absence of lung inflammation. | Aged SH rats (12-13 months)  Adult SH rats (6-7 months) | Upadhyay et al (2014)  Upadhyay et al (2008) |
| Concentrated air particles (CAPs),  Raleigh-Durham (USA), < 2.5 µm | 100-2,758 µg/m^3^) for 4 h/day, 2 days | BAL neutrophil increase in all models and exposures. | SH and WKY rats | Kodavanti et al (2005) |
| Concentrated air particles (CAPs),  Detroit (USA), < 2.5 µm | 103-918 µg/m^3^) (mean = 502 µg/m^3^, 8 h/d for 13 consecutive days. | Mild pulmonary and systemic changes; increase of metals in the lung. | SH and WKY rats | Rohr et al (2010) |
| CAPS versus diesel exhaust particles (DEPs) | 30 or 300 µg/m^3^ DEPs,  356 and 595 µg/m^3^ CAPs | Enhancement of allergic airway disease by CAPs. | Ovalbumin-challenged Brown Norway (BN) rats | Harkema et al (2009) |

**Supplementary Table 2.** Comparison of outcomes of inhalation studies with microplastic (MP) and particulate matter (PM) in normal and compromised animals.
